# ACC-1 as a Possible Biochemical Indicator of Lipoapoptosis in In Vivo and In Vitro Models of MAFLD

**DOI:** 10.3390/ijms26083459

**Published:** 2025-04-08

**Authors:** David Ibarra Martínez, Israel Alejandro Muñoz Nieto, David Alejandro Hernández Marín, Javier Ventura Juárez, Sandra Luz Martínez Hernández, Esperanza Sánchez Alemán, Raquel Guerrero Alba, Martín Muñoz Ortega

**Affiliations:** 1 Departamento de Química, Universidad Autónoma de Aguascalientes, Aguascalientes 20100, Mexico; david.ibarra@edu.uaa.mx (D.I.M.); alex_defiler@hotmail.com (I.A.M.N.); 2 Departamento de Microbiología e Inmunología, Universidad Autónoma de Aguascalientes, Aguascalientes 20100, Mexico; david.hernandezm@edu.uaa.mx (D.A.H.M.); luz.martinez@edu.uaa.mx (S.L.M.H.); 3 Departamento de Morfología, Universidad Autónoma de Aguascalientes, Aguascalientes 20100, Mexico; javier.ventura@edu.uaa.mx (J.V.J.); esperanza.sanchez@edu.uaa.mx (E.S.A.); 4 Departamento de Fisiología y Farmacología, Universidad Autónoma de Aguascalientes, Aguascalientes 20100, Mexico; raquel.guerrero@edu.uaa.mx

**Keywords:** NAFLD, ACC-1, biomarker, lipocytotoxicity, hepatic steatosis, steatohepatitis

## Abstract

Non-alcoholic fatty liver disease (NAFLD) is an emerging condition with a worldwide prevalence ranging from 6% to 35% and is very frequent among patients with obesity, diabetes, or metabolic syndrome. One of the main challenges in the treatment of this disease is the identification of a reliable and direct biomarker to diagnose the stage of hepatic steatosis before it progresses to steatohepatitis. This is especially important as many patients remain asymptomatic until cirrhosis develops. The aim of this study was to analyze the expression of the enzyme acetyl-CoA carboxylase 1 (ACC-1) in vitro in a model of lipocytotoxicity using HepG2 cells as well as in vivo in Wistar rats. Our results demonstrate an accumulation of lipid inclusions in hepatocytes observed both in vitro and in experimental models of hepatic steatosis, leading to membrane damage. This allows for the detection of ACC-1 enzyme in the extracellular medium at short induction times, in contrast to the appearance of AST and ALT, which become detectable only once the damage becomes more invasive. ACC-1 could potentially serve as a clinical indicator to detect fatty liver disease before it progresses to steatohepatitis and fibrosis, allowing for timely and non-invasive treatment for patients.

## 1. Introduction

Hepatic steatosis, or non-alcoholic fatty liver disease (NAFLD), refers to the accumulation of fatty acids and triglycerides in hepatocytes. It primarily affects individuals with very low or virtually no alcohol consumption. This disease is commonly observed in people suffering from obesity, type II diabetes mellitus, hypertriglyceridemia, and those taking medications that can induce hepatic steatosis. NAFLD is the most prevalent liver disease worldwide, affecting nearly one-third of the global population. It has become the leading cause of liver transplants in Mexico, where various risk factors for the disease are prevalent, and its prevalence may exceed 50% [1,2].

Without proper monitoring and with continuous chronic damage, this condition leads to inflammation, permanent liver damage, fibrosis, and, in extreme cases, hepatocellular carcinoma. NAFLD is closely associated with insulin resistance syndrome and hyperglycemia. This phenomenon promotes the accumulation of free fatty acids in the liver, which, within hepatocytes, act as substrates and inducers of mitochondrial cytochrome lipoxygenases. This process increases the levels of cytochrome CYP2E1 and, consequently, boosts the production of free radicals. These radicals contribute to oxidative stress and enhance lipid peroxidation in hepatocyte membrane systems, resulting in progressive liver damage [1,3,4,5].

The observed damage is chronic due to lipid accumulation, which, in the long term, causes mitochondrial dysfunction resulting from excess glucose. This leads to the generation of reactive oxygen species (ROS), which, together with accumulated lipids, cause the peroxidation of membrane systems. The pro-apoptotic protein BAX facilitates the release of cytochrome C from the mitochondria, regulated by BCL-2. If this regulation is absent, cytochrome C leaks into the cytoplasm, triggering a caspase cascade and initiating cellular apoptosis. This cascade, combined with the persistent damage to the membrane, leads to cell death and the release of enzymes into the extracellular space [3,6].

For diagnosing hepatic steatosis, which presents a silent and challenging histopathological scenario, recent methods have been proposed to non-invasively determine a range of parameters for timely identification of hepatic steatosis and non-alcoholic steatohepatitis. The goal is to prevent the progression to hepatic fibrosis. However, parameters such as body mass index (BMI), waist circumference, and triglyceride levels are unreliable when assessing the different stages of steatosis. More accurate methods, such as liver fat scoring using magnetic resonance imaging (MRI), offer improved precision. However, the use of MRI is not commonly practiced [1,2,3,4,5,6,7].

The SteatoTest evaluates a broader range of parameters compared to the FibroTest, including α2-macroglobulin, haptoglobin, apolipoprotein A1, GGT, total bilirubin, and ALT, as well as variables such as BMI, cholesterol, triglycerides, and glucose. While its effectiveness is superior, the test has significant limitations, such as its inability to differentiate between the various stages of steatosis. Furthermore, the inclusion of multiple tests in a single examination makes it a costly option, reducing its clinical feasibility [1,7].

Currently, various biomarkers are available to assess the damage and stage of steatosis. However, many of these biomarkers suffer from significant limitations, particularly regarding cost and their inability to detect early-stage damage. This hinders timely diagnosis and complicates the prevention of further liver damage [7,8,9].

Acetyl-CoA carboxylase is an enzyme that catalyzes the carboxylation of acetyl-CoA to produce malonyl-acetyl-CoA. There are two main isoforms, ACC1 and ACC2 (acetyl-CoA carboxylase-1 and -2), which are found in higher proportions in tissues such as adipose tissue and the liver. ACC1, located in the cytosol, regulates de novo lipid synthesis (lipogenesis), while ACC2, located on the outer mitochondrial membrane, plays a key role in fatty acid oxidation by regulating carnitine palmitoyltransferase 1 (CPT1) [10,11].

At present, treatments aimed at blocking or inhibiting the expression of FASN (fatty acid synthase) and ACC have been investigated, showing results ranging from reduced de novo lipogenesis to weight loss in patients. Treatments such as metformin, which also helps to regulate type II diabetes, along with GS-0976 and MK-4074, potent inhibitors of ACC-1 and ACC-2, have advanced the understanding of the crucial role that these enzymes play in regulating NAFLD (non-alcoholic fatty liver disease) [12,13,14]. In vivo studies have yielded promising results, suggesting that managing NAFLD with a focus on ACC and its isoforms could be critical in preventing progression to non-alcoholic steatohepatitis [15]. Moreover, in vitro assays have demonstrated the impact of ACC-1 in a lipotoxicity model, a form of cellular damage associated with NAFLD [16]. Based on these findings, in which ACC-1 is identified as a potential treatment target, the present research explores this enzyme as a promising candidate for early diagnosis of NAFLD, both in vitro and in vivo.

## 2. Results

### 2.1. Effect on the Viability of HepG2 Liver Cells During Their Interaction with the High-Calorie Medium

**Figure 1:**
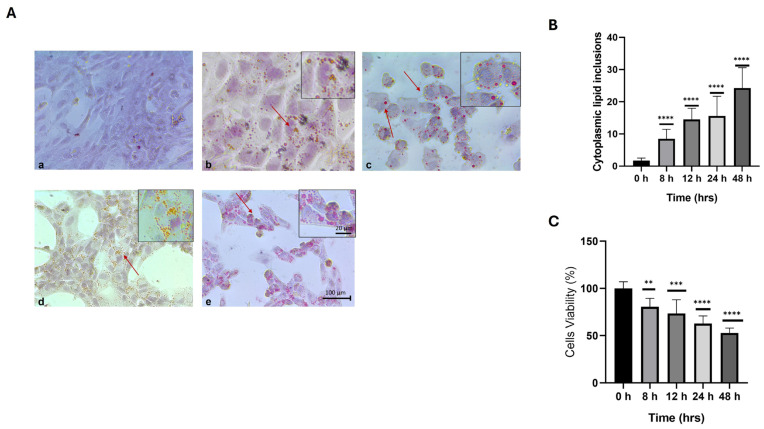
Oil Red O staining technique (Panels **A**–**C**) reveals a time-dependent increase of lipid inclusions in cells cultured in hypercaloric medium with respect to the control (**a**). At 8 h (**b**), a significant increase in the number of lipid inclusions is observed (arrows red). Subsequently, a gradual increase in the number of inclusions is observed at 12, 24, and 48 h (**c**–**e**), Panel (**B**), as well as a decrease in cell viability (Panel **C**) and morphological changes in liver cells. Data are presented as mean  ±  SEM. Statistically significant differences between 0 h and treatments are indicated ** *p*  <  0.01, *** *p*  <  0.001, and **** *p*  <  0.0001.

The Oil Red O staining technique (Panel A) revealed a time-dependent increase in lipid inclusions in cells cultured in the high-calorie medium (Figure 1). No inclusions were observed in the control group (a). After 8 h of treatment, small cytoplasmic lipid inclusions began to appear; however, the characteristic cell morphology was maintained (b). At 12 h, significant changes were evident, including noticeable deformation of the cell morphology, with the nucleus shifting to the periphery due to the accumulation of cytoplasmic lipids and cellular ballooning (c). At 24 h, there were marked increases in inclusions, cellular deformation, and loss of monolayer integrity (d). After 48 h, cellular deformation, lipid inclusions, cellular remnants, and a reduced number of cells were observed (e). Additionally, cell viability was assessed using the MTT assay during the interaction of HepG2 cells with the high-calorie medium at 8, 12, 24, and 48 h. A time-dependent decrease in cell viability was observed, which was inversely proportional to the increase in lipid inclusions in the cytoplasm. The aim of this analysis was to determine the time at which cell viability began to be affected in relation to the increase in intracellular lipid inclusions. Figure 1, Panels B and C.

### 2.2. Effect of the Hypercaloric Medium on Monolayer Integrity and Membrane Permeability in HepG2 Cells

**Figure 2:**
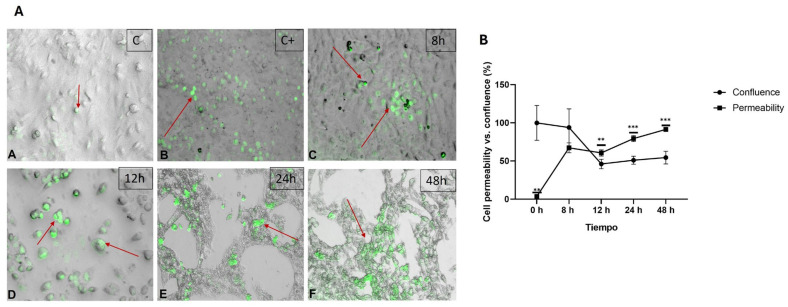
Interaction of liver cells with high-calorie medium increases cell permeability. Panel (**A**) shows an increase in fluorescence (arrows red) with respect to time. In the control, minimal fluorescence is observed, and the integrity of the monolayer is maintained (**A**). In the positive control exposed to peroxide (1 µL), increased fluorescence is observed compared to the negative control, indicating significant cell membrane damage (**B**). At 8 h, the morphological integrity of the cells is maintained (**C**). After 12, 24, and 48 h, the integrity of the monolayer is affected and the fluorescence intensity increases, with a higher number of permeabilized cells and, consequently, possible cell lysis (**D**–**F**). In panel (**B**), it is observed that as the integrity of the monolayer decreases, reflected in the percentage of confluence, the percentage of permeability increases in HepG2 cells exposed to the hypercaloric medium. Data are presented as mean  ±  SEM. Statistically significant differences between 0 h and treatments are indicated ** *p*  <  0.01, *** *p*  <  0.001.

In the SYTOX Green staining, Figure 2 Panel A shows an increase in fluorescence over time. In the control, minimal fluorescence was observed, and the monolayer integrity was maintained (a). In the positive control exposed to peroxide (1 µL), higher fluorescence was seen compared to the negative control, indicating significant damage to the cell membrane (b). At 8 h, the morphological integrity of the cells remained, and the monolayer was partially intact; however, some cells began to exhibit faint fluorescence, indicating membrane permeabilization and compromised viability (c). After 12 h, the monolayer integrity was affected, and fluorescence intensity increased, with more cells becoming permeabilized and consequently damaged (d). After 24 h, the most significant changes occurred, including cellular destruction, near-total loss of the monolayer, and a further increase in both the quantity and intensity of fluorescence (e). At 48 h post-treatment, minimal cellular recovery was observed, with higher fluorescence intensity and cellular remnants present (f). In Figure 2 Panel B, it is observed that as the monolayer integrity decreases, reflected in the percentage of confluence, the percentage of permeability increases in HepG2 cells exposed to the high-calorie medium, suggesting that there was initial membrane damage at 8 h, followed by progressive deterioration.

### 2.3. Lipoapoptotic Effect of the Hypercaloric Medium on HepG2 Cells

**Figure 3:**
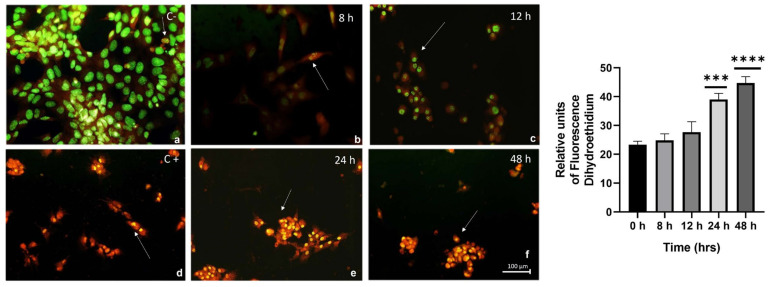
Effect of high-calorie medium on liver cells, from apoptosis to the necrotic process, as assessed by Acridine Orange staining (arrows). The figure shows the contrast between the negative damage control (**a**) and the positive control using hydrogen peroxide (**d**). During the experiment, a slight loss of morphology is observed, which can still be seen at 8 and 12 h (**b**,**c**). At 24 h, late apoptosis is observed, with an increase in orange staining of phagocytic structures, although certain cellular features are still preserved (**e**). At 48 h, orange staining is evident throughout the cell in that field, along with cellular depletion possibly due to the necrotic process (**f**). Dihydroethidium staining shows increasing fluorescence in cells over time, depending on the duration of exposure and the number of hours the cells are in contact with the hypercaloric medium. Data are presented as mean  ±  SEM. Statistically significant differences between 0 h and treatments are indicated *** *p*  <  0.001, and **** *p*  <  0.0001.

Acridine Orange fluorescent dye is useful for detecting structures such as DNA and chromosomes, which stain green, indicating active cell division. Similarly, RNA-rich and highly acidic structures, such as lysosomes and phagolysosomes, stain orange. This approach allows the technique to not only differentiate between these two structures but also to distinguish healthy cells from cells in the apoptotic phase. In Figure 3, the control shows typical DNA staining in cells with active cell division and no apparent damage (A). After 8 h, partial orange staining and green nuclei were seen, indicating the presence of early apoptosis in the cell (B). After 12 h of exposure to the high-calorie medium, green-stained nuclei and orange-stained cytoplasm were observed, along with a slight loss of morphology that can still be appreciated at 8 h (C). At 24 h, late apoptosis was observed, with enhanced orange staining of phagocytic structures, although certain cellular features were still preserved (E). After 48 h, orange staining was evident throughout the entire cell in that field, along with cellular decrease and aggregates, as seen in the SYTOX Green and Oil Red O staining (F). The positive control was similar to the 24 h time point, as it retained certain morphological characteristics, compared to the 48 h time point, where only cellular aggregates were visible (D).

Induced by mitochondrial stress due to increased metabolism, mitochondrial oxidative stress is heightened, leading to the generation of free radicals, primarily the superoxide anion. Dihydroethidium staining showed increasing fluorescence in cells over time, depending on the duration of exposure and the number of hours the cells were in contact with the high-calorie medium (Figure 3). This resulted in membrane damage caused by lipoperoxidation and, subsequently, cell death.

### 2.4. Detection of ACC-1 in Early Stages of Cellular Damage Compared to Hepatic Enzymes AST and ALT

**Figure 4:**
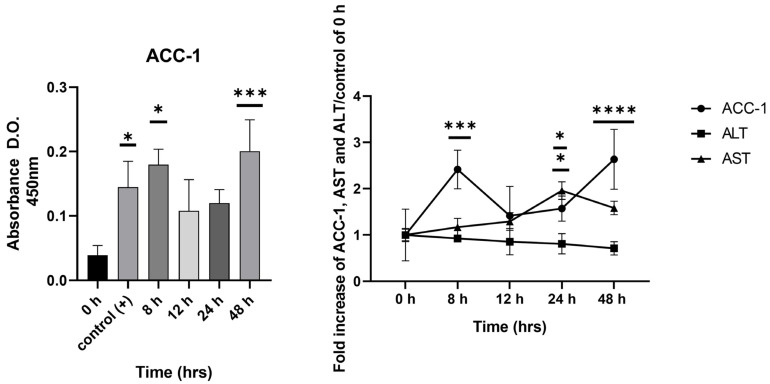
The detection of ACC-1 in the extracellular medium is sensitive from 8 h of interaction of liver cells with high-calorie medium. This increase is observed at 8 h, which then decreases at 12 h and increases again from 24 h to 48 h, reaching its peak. In the case of AST, an increase is detected at later time points, with its peak occurring at 24 h of exposure to the hypercaloric medium, indicating that the ACC-1 biomarker is temporally detected when mild damage occurs in the monolayer, compared to transaminases, which are detected when more advanced damage occurs in the monolayer. In the case of ALT, no significant changes are observed at different incubation times with the hypercaloric medium. The positive control was total lysate from hepG2 cells. Data are presented as mean  ±  SEM. Statistically significant differences between 0 h and treatments are indicated * *p*  <  0.05, *** *p*  <  0.001, and **** *p*  <  0.0001.

Using the ELISA technique, an increase in ACC-1 released into the extracellular medium was detected in cells cultured for short periods of interaction with the hypercaloric medium. This increase was observed at 8 h, which then declined at 12 h and increased again from 24 h to 48 h, reaching its peak. For AST, an increase was detected at later time points, with its peak occurring at 24 h of exposure to the hypercaloric medium, indicating that the ACC-1 biomarker was temporally detected when mild damage to the monolayer occurred, compared to transaminases, which are detected when more advanced damage to the monolayer occurs (Figure 4). In the case of ALT, no significant changes were observed at the different incubation times with the hypercaloric medium.

### 2.5. Expression of Cellular Stress Markers and Lipogenic Enzymes over the Course of Interaction with the Hypercaloric Medium

**Figure 5:**
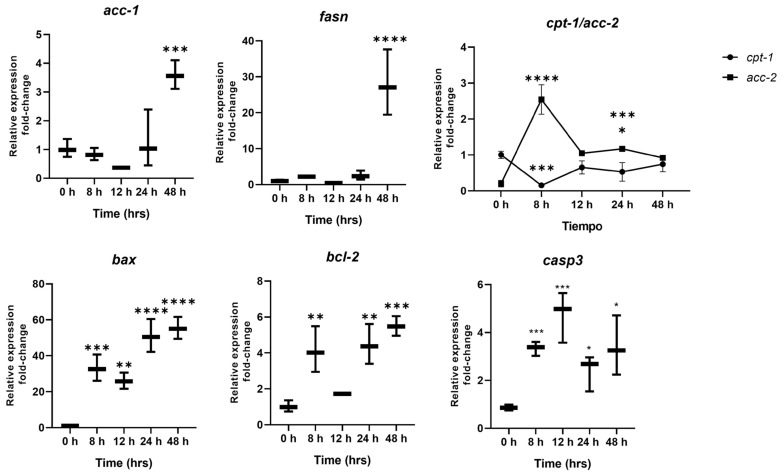
Regulation of the expression of different metabolic and survival genes in HepG2 cells interacting with high-calorie medium. Significant increases in *acc-1* and *fasn* expression can be observed at 48 h. In terms of regulation, there is no significant activity of *cpt-1*, which is regulated by *acc-2*. On the other hand, the expression of bax is significantly increased, possibly caused by mitochondrial stress from 8 h, as well as that of *bcl-2*, which tries to regulate cellular stress. Finally, *caspase 3* expression tends to decrease, possibly due to the change from an apoptotic to a necrotic process. Data are presented as mean ± SEM. Statistically significant differences between 0 h and treatments are indicated * *p* < 0.05, ** *p* < 0.01, *** *p* < 0.001, and **** *p* < 0.0001.

This section (Figure 5) aims to compare different markers activated during cellular damage in relation to the gene expression of *acc-1* and the identification of ACC-1 by ELISA, which is detected through membrane permeability resulting from cellular damage. Therefore, this section infers the sensitivity of detecting our biomarker at early stages of cellular damage in comparison to other biomarkers. The *acc-1* and *fasn* expression levels were low at early time points, possibly due to the presence of preformed enzymes in the cytoplasm; however, peaks in *acc-1* and *fasn* expression were observed at 48 h, likely due to the continued influx of glucose into the hepatic cell, maintaining the lipogenic state. This correlated with the increase in *acc-2* expression relative to *cpt-1* expression, an important enzyme in fatty acid catabolism. On the other hand, the expression levels of *bax* and *caspase 3* rose at 8 h in the monolayer cells due to mitochondrial stress. For *bcl-2*, high expression was also visible at 8 h, maintained until 12 h, and increased at 48 h, indicating a possible regulation of apoptotic pathways without a significant effect. The expression of *caspase 3* tended to increase at the short time of 8 h, remained steady, and then decreased at 48 h, possibly indicating a shift from apoptosis to necrosis, as observed in Figure 1, Figure 2 and Figure 3 (Oil Red O, Sytox, and Acridine Orange at 48 h), where cell confluence decreased.

### 2.6. The Hypercaloric Diet Resulted in an Increase in Weight and Alteration of Metabolic Biochemical Parameters and Liver Damage Markers

**Figure 6:**
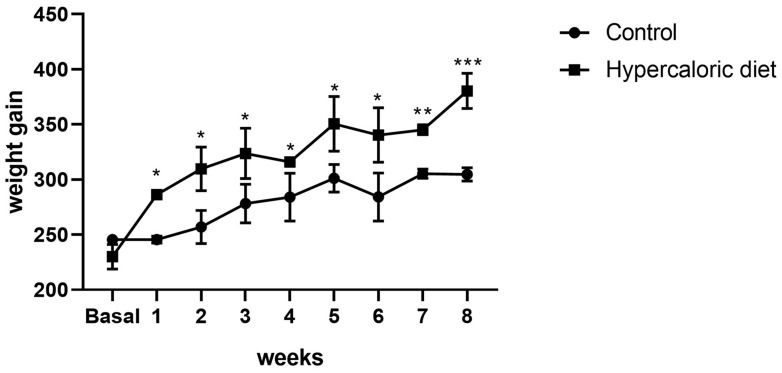
Increase in body weight of rats treated with the high-carbohydrate and high-fat hypercaloric diet relative to rats treated with the standard diet for 8 weeks. * *p* < 0.05, ** *p* < 0.01 and *** *p* < 0.001.

**Figure 7:**
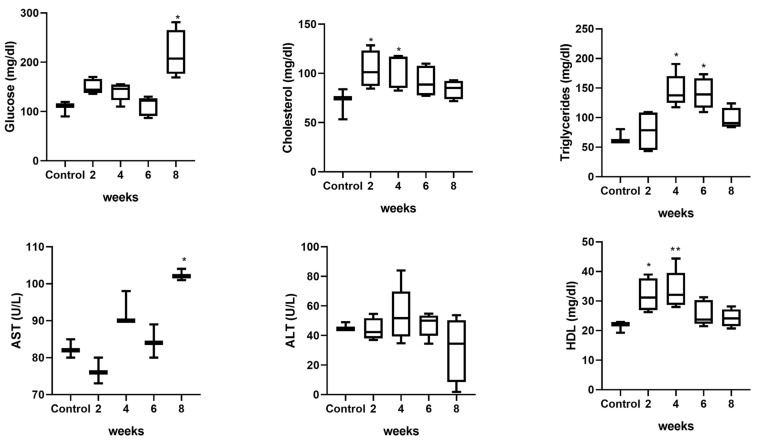
Effect of the high-calorie diet on Wistar rats. Hyperlipidemia is observed between weeks 2 and 6 and, subsequently, hyperglycemia is observed at 8 weeks relative to the control group. Finally, regarding liver damage enzymes, AST and ALT, a significant increase in AST levels is observed at 8 weeks compared to the control group, with no statistical significance found at weeks 2, 4 and 6, nor with ALT levels. Data are presented as mean ± SEM. Statistically significant differences between control and treatments are indicated * *p* < 0.05, ** *p* < 0.01.

After administering the hypercaloric diet designed by our laboratory ad libitum to the rat group to induce fatty liver and obesity, we initially observed a significant increase in body weight in the rats fed the high-fat and carbohydrate diet compared to the basal control group, with the most pronounced significance at 8 weeks of daily administration (Figure 6). Regarding metabolic biochemical parameters, a significant increase in serum glucose levels was observed at 8 weeks of high-calorie diet administration compared to the control group (*p* < 0.05). Furthermore, levels of triglycerides, HDL, and cholesterol were significantly higher at weeks 2, 4, and 6. Lastly, concerning liver damage enzymes, AST and ALT, a significant increase in AST levels was observed at 8 weeks compared to the control group, with no statistical significance found at weeks 2, 4, and 6, nor with ALT levels (Figure 7).

### 2.7. Histopathological Changes Induced by the Hypercaloric Diet and Early Identification of the ACC-1 Enzyme in the Serum of Experimental Animals

**Figure 8:**
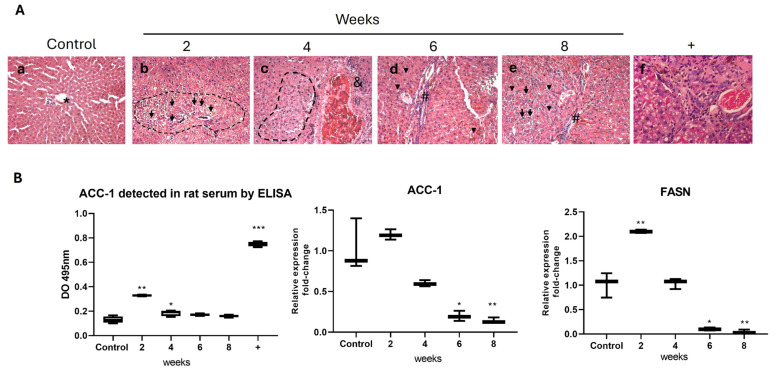
Panel (**A**): Different histological alterations in the livers of rats under a hypercaloric diet are observed with respect to the evaluation time: (**a**) Control rat liver with normal hepatic structure, showing an organized distribution of the parenchyma surrounding the central venule (asterisk). (**b**) Ballooning of hepatocytes (arrow), observed focally in the hepatic parenchyma. (**c**) Perivascular thickening with possible inflammatory infiltrate into the hepatic parenchyma (&) and disorganization of the cellular architecture (dashed line). (**d**) Presence of perivascular fibrosis (#), subtle microvesicular steatosis (arrowhead). (**e**) Hepatocyte ballooning, fibrosis, and steatosis. (**f**) Positive control for liver damage, with thickening of the vascular wall, steatosis, inflammation, and marked fibrosis (Animal treated with CCl4 0.8 mL/kg). Panel (**B**): Presence of ACC-1 in the serum of rats treated with the hypercaloric diet, a significant increase was observed at 2 and 4 weeks compared to the intact control. Regarding *acc-1* and *fasn*, their expression increased at 2 weeks of the diet. Data are presented as mean ± SEM. Statistically significant differences between control and treatments are indicated * *p* < 0.05, ** *p* < 0.01, and *** *p* < 0.001.

Panel A, Figure 8: Different histological alterations in the livers of rats under a hypercaloric diet are observed with respect to the evaluation time: (A) Control rat liver with normal hepatic structure, showing an organized distribution of the parenchyma surrounding the central venule (asterisk). (B) Ballooning of hepatocytes (arrow), observed focally in the hepatic parenchyma, primarily in zone 3 of the acini, possibly caused by oxidative stress. (C) Perivascular thickening with possible inflammatory infiltrate into the hepatic parenchyma (&) and disorganization of the cellular architecture (dashed line). (D) Presence of perivascular fibrosis (#), subtle microvesicular steatosis (arrowhead), and inflammation in the hepatic tissue. (E) Hepatocyte ballooning, fibrosis, and steatosis. (F) Positive control for liver damage, with thickening of the vascular wall, steatosis, inflammation, and marked fibrosis (animals treated with CCl4 0.8 mL/kg) (Figure 8). The images reveal the progression of liver damage, from cellular changes to fibrosis, under the hypercaloric diet.

Panel B, Figure 8: Once the presence of the ACC-1 enzyme was determined using the ELISA technique in the serum of rats treated with the hypercaloric diet, a significant increase in the ACC-1 level was observed at 2 and 4 weeks compared to the intact control, consistent with the maximum increases in triglycerides and cholesterol. Subsequently, the detected levels of ACC-1 decreased at 6 and 8 weeks of treatment with the hypercaloric diet. In the positive control, an increase consistent with liver damage was observed. Regarding *acc-1* and *fasn*, their expression increased at 2 weeks of the diet, decreasing at week 4 and attenuating by 8 weeks of the hypercaloric diet.

## 3. Discussion

NAFLD continues to pose a significant challenge, largely due to the stages of the disease. Once fibrosis begins, the progression of liver damage accelerates [4,17,18]. Currently, treatments aimed at blocking or inhibiting the expression of FASN, ACC-1, ACC-2, and metalloproteinase-1 have been investigated, with the results showing a reduction in de novo lipogenesis and weight loss in patients. Treatments such as metformin, which also aids in the regulation of type 2 diabetes, as well as GS-0976 and MK-4074—potent inhibitors of ACC-1 and ACC-2—help to better understand the critical role that these enzymes play in the management of patients with NAFLD [12,13,14].

On the other hand, in vivo studies have shown promising results, demonstrating that targeting NAFLD by focusing on ACC and its isoforms could play a crucial role in preventing the progression of the disease to non-alcoholic steatohepatitis [15]. Based on these reports, where ACC-1 is proposed as a potential therapeutic target, the current study investigated ACC-1 as an alternative for early diagnosis of NAFLD. Using both in vitro and in vivo assays, the influence of ACC-1 was demonstrated in a model of hepatotoxicity, a form of cellular damage associated with NAFLD [16]. In this way, using an established in vitro model of lipotoxicity in HepG2 cells, as well as an animal model of fatty liver, the enzyme ACC-1 was identified at the serum level using the ELISA technique during the early stages of cellular and tissue damage, such as necrosis and fibrosis, respectively.

To establish the hepatotoxicity model, initial experiments using MTT and Oil Red O assays demonstrated a gradual reduction in cell viability, starting at around 20% after 8 h of exposure to the high-calorie medium and reaching 50% at 48 h. This decrease in viability strongly correlated with the increase in cytoplasmic lipid inclusions, similar to the findings reported by Jang et al. [19], who observed a gradual decline in viability starting at one hour and continuing up to 24 h of exposure to hypercaloric medium, with an increase in lipid inclusions at 24 h. By contrast, our results showed that these lipid inclusions were observed as early as 8 h after treatment. By 12 h, typical morphological changes were noted, resembling those seen in patients with NAFLD, such as nuclear displacement toward the periphery and apoptotic features [16]. These findings further confirmed the validity of our experimental model.

Using SYTOX Green stain, dye infiltration into the cells was observed at different time points, indicating a loss of cell membrane integrity (membrane permeability damage) and resulting in green fluorescence. This morphological finding has been reported in patients with NAFLD, reflecting the release of cytoplasmic contents from cells damaged by oxidative stress [6,20]. When HepG2 cells were exposed for periods longer than 24 and 48 h, an increase in dye infiltration was observed compared to shorter time points (8 and 12 h), highlighting the progressive membrane damage caused by exposure to the high-calorie medium. Additionally, morphological changes and the presence of cellular remnants were identified, as observed by Medina Pizaño et al. [20].

It is well established that there is a strong correlation between the increase in oxidative stress and apoptosis in patients who progress from NAFLD to NASH [6]. We assessed the level of oxidative stress induced by the high-calorie medium in liver cells, which was associated with an increase in apoptosis and loss of the cellular monolayer. Detection of superoxide radicals using the dihydroethidium technique in HepG2 cells treated with the high-calorie medium revealed a time-dependent increase, supporting the findings obtained with Acridine Orange staining. This staining highlighted an apoptotic process, and in line with the SYTOX Green results, it may suggest the possible activation of cellular necrosis due to persistent oxidative stress and membrane damage.

With continuous exposure to the high-calorie medium, increases in the expression of *acc-1* and *fasn* were observed. This is because prolonged exposure of HepG2 cells to high concentrations of glucose and fatty acids activates the de novo lipid synthesis pathway [10,21], resulting in increases in the expression of *acc-1* and *fasn* between 24 and 48 h. The fatty acids generated both through de novo synthesis and those directly entering from the high-calorie medium blocked the transporter enzyme CPT1, thus regulating malonyl-CoA levels [11]. By blocking the entry of fatty acids into β-oxidation, these accumulate in the cytoplasm [6,22].

Mitochondrial stress, resulting from the excess lipids derived from the high-calorie medium and glucose, which ultimately leads to de novo lipogenesis, generates superoxide radicals. These radicals promote the expression of pro-apoptotic genes like *casp3* and *bax*, which are regulated by *Bcl-2* [6,21]. Both *casp3* and *bax* are associated with mitochondrial stress [23,24]. While it is well known that mitochondrial damage can trigger apoptotic pathways [25,26], our results showed that *Bcl-2* was unable to control the damage caused by the high-calorie medium, confirming the damage to HepG2 cells.

Once the damage induced by the high-calorie medium was demonstrated, evidenced by the reduction in cell populations, lipid accumulation, and membrane damage due to lipocytotoxicity, the presence of the enzyme ACC-1 in the extracellular medium, specifically in the culture medium, was assessed using the ELISA technique [27,28]. The increase in optical density at 450 nm, resulting from the antibody peroxidation reaction, showed an increase starting at 8 h, linked to cell permeability. Subsequently, the optical density decreased between 12 and 24 h, and then rose again at 48 h. This pattern suggested that ACC-1 was released outside of the cell during the early stages of damage induction and continued to be released throughout the evaluated time period. The observed decreases in enzyme levels at 12 and 24 h may have been related to a deficit in cellular function, as reported in some biochemical biomarkers associated with chronic damage [9].

To compare the detection of this enzyme as an indicator of cellular damage, the transaminases ALT and AST, commonly used as biomarkers of liver damage, were evaluated [4,22]. These transaminases were detected in the culture medium at 24 and 48 h of exposure to the high-calorie medium. Additionally, in the in vivo model, it was demonstrated that the high-calorie diet increased body weight compared to the control group, along with an increase in metabolic parameters such as glucose, triglycerides, and cholesterol, indicating hyperlipidemia.

In the in vivo model, as in the in vitro model, a serum increase in the enzyme ACC-1 was observed at early stages of liver damage induction, specifically at 2 and 4 weeks. This increase was closely correlated with the hyperlipidemia observed in the experimental groups, suggesting that ACC-1 could serve as an early biomarker for detecting cellular damage. Similarly, Rameshreddy et al. [29] reported an increase in ACC-1 expression, linked to hyperlipidemia in experimental animals, a finding that aligned with the results obtained in our study at 2 weeks following high-calorie diet administration.

It is important to note that, after the second week, ACC-1 levels progressively decreased, which could be related to adaptive mechanisms in the liver, such as the activation of AMPK, as described by Hernández-Puga et al. [30]. AMPK inhibits ACC and promotes fatty acid oxidation to help mitigate metabolic damage.

The alterations observed in *acc-1* and *fasn* at the molecular level (PCR), where both showed decreases in expression, could also be explained by the activation of AMPK. Pang et al. [31] emphasized that AMPK functions as a master metabolic sensor, regulating energy homeostasis through the phosphorylation of key enzymes such as ACC. This mechanism not only reduces fatty acid synthesis but also promotes lipid oxidation under conditions of metabolic stress.

Hernández-Puga et al. [30] described that the activation of AMPK, induced by an increase in the AMP/ATP ratio, serves as a critical protective mechanism against excessive lipid accumulation and liver damage. Additionally, Neuman et al. [4] highlighted that AMPK activation may help to prevent the progression of non-alcoholic fatty liver disease by limiting hepatic triglyceride accumulation.

Recent studies suggest that glucose metabolism occurs to a greater extent in patients with NAFLD than in healthy individuals. Adipocytes show an increased capacity to handle and store triglycerides during critical situations through the generation of new cells [32]. ACC-1 plays a crucial role in this process as an energy-regulating enzyme. Understanding the functional role of these enzymes, their interactions with other enzymes, their regulation, and their potential as therapeutic targets is key to providing a clearer understanding of NAFLD and its progression to non-alcoholic steatohepatitis (NASH) [14,33].

In line with the research data, the introduction of glucose into the cell activates the de novo lipid synthesis pathway. Additionally, the fatty acids from the medium that enter the cell directly contribute to the formation of lipid inclusions. Meanwhile, fatty acid β-oxidation is blocked by the regulator malonyl-CoA, which inhibits CPT1 [14]. The constant accumulation of lipids is intentional, as it simulates the conditions of a patient with NAFLD [34,35]. Through mitochondrial stress, superoxide radicals are generated, which, via lipid peroxidation, damage the membrane, releasing ACC-1 into the extracellular medium. Similarly, the activation of apoptosis causes further cellular damage.

## 4. Strengths, Limitations, and Perspectives

According to our in vitro studies, ACC1 could be a potential clinical marker for patients with NAFLD, as it is detectable at early stages when cellular viability is not yet significantly affected by oxidative stress and cellular permeability, in contrast to AST, which is detected when cellular viability is already compromised. On the other hand, in the animal model, ACC1 is detected in the presence of hyperlipidemia, making it sensitive to metabolic alterations. However, we found that its level declined in the samples, which could affect the timing of its identification. Further research is needed to better understand the factors that influence changes in ACC1 release due to early membrane damage and permeability.

## 5. Materials and Methods

### 5.1. Cell Culture

HepG2 cells (donated from the immunoparasitology laboratory, Universidad Autónoma de Aguascalientes) were grown in DMEM F12 (D5546; Sigma, STL, Saint Louis, MO, USA) supplemented with 2% fetal bovine serum (SU-420, microlab), 2% L-glutamine (25-005-CI; Corning, Corning, NY, USA), and 1% penicillin/streptomycin (30-001-CI; Corning) at 37 °C in a humidified atmosphere containing 5% CO_2_ and 95% O_2_, as proposed by ATCC.

### 5.2. Induction of Lipotoxicity in HepG2 Cells and MTT Cell Viability Assay

For the lipotoxicity protocol, 5 × 10^3^ cells per well were initially incubated in supplemented DMEM F12 at 37 °C under 5% CO_2_ for 24 h. After this time, the DMEM F12 medium was removed and replaced with low-glucose DMEM (5.5 mM; D2902; Sigma), without supplementation, for 24 h (starvation period). Once the time had elapsed, the low-glucose medium was removed and replaced with the hypercaloric medium, which contained DMEM F12 and NEFA 0.5 mM (oleic acid 0.33 mM and palmitic acid 0.17 mM, O1383,P0500; Sigma) [19], followed by incubation at 37 °C under 5% CO_2_ for 8, 12, 24, and 48 h. At the end of each time point, the medium was removed, and 100 µL of MTT (0.5 mg/mL, Sigma M5655) was added to each well and incubated under the same conditions for 4 h. After incubation, the MTT was removed, and the formed crystals were dissolved with 100 µL of DMSO (Sigma, D8418), followed by reading the absorbance at 595 nm. Each experiment was performed in triplicate (*n* = 3) to ensure the reproducibility of the results.

### 5.3. Oil Red O Staining

Cells for lipid droplet staining were fixed for 15 min in 4% paraformaldehyde. Oil-Red O (0.5% *w*/*v*, Sigma, 00625) in isopropanol was added to the cells for 15 min. The Oil Red O solution was removed, and the cells were washed for 5 min with dH_2_O. The cells were then counterstained with hematoxylin. Phase-contrast images were obtained using an OptikamB10 digital camera attached to an IM-3 Series microscope. Each experiment was performed in quadruplicate (*n* = 4) to ensure the reproducibility of the results.

### 5.4. SYTOX Green Staining

The HepG2 cells treated with hypercaloric medium were fixed on a coverslip and washed with 1× PBS. A positive damage control was prepared by adding 250 μM of 30% hydrogen peroxide for 15 min. SYTOX^®^ Green nucleic acid stain (Invitrogen, Thermo Fisher Scientific, Waltham, MA, USA ) solution was prepared with stock solution diluted to 1:30,000 (167 nM) in 1× PBS. A volume of 500 μL of the staining solution was used to cover the cells, which were then incubated in the dark for 17 min at 37 °C. After incubation, the cells were washed with 1× PBS and mounted on coverslips with glycerol gel. The images were analyzed at a magnification of 10× using a Carl Zeiss 398 Axiovert 40CFL fluorescence inverted microscope (Carl Zeiss AG, Oberkochen, Germany) with a maximum emission of 504–523 nm. Each experiment was performed in quadruplicate (*n* = 4) to ensure the reproducibility of the results.

### 5.5. Acridine Orange (AO) Staining

To prepare the AO (Sigma, A6014) staining solution, the stock solution was prepared by dissolving 1 g of AO in 100 mL of 1× PBS (pH 7.4), which was stored at 4 °C away from light [36]. HepG2 cells (10^5^), which were treated with hypercaloric medium, were stained with 1 µg/mL AO. After incubation for 15 min at 37 °C, the stained cells were washed with 1× PBS. The presence of orange staining due to possible cell damage was evaluated using an inverted fluorescence microscope (Carl Zeiss 398 Axiovert 40CFL Microscope; Carl Zeiss AG, Oberkochen, Germany) at a magnification of 200×, with a maximum emission of 490 nm in red and 515 nm in green. Each experiment was performed in quadruplicate (*n* = 4) to ensure the reproducibility of the results.

### 5.6. Evaluation of Oxidative Stress Using Dihydroethidium (DHE)

In total, 250,000 cells per well were seeded in a 12-well plate and treated, as previously described, to obtain the kinetic data for cells with lipotoxicity. At the end of the time points, the cells were scraped, centrifuged in Eppendorf tubes at 3000 rpm for 5 min, and the supernatant was removed. The cells were washed with 1× PBS, and finally, 500 µL of HEPES sodium buffer pH 7.2 and 0.2 μM DHE (Life Technologies, D11347, Carlsbad, CA, USA) were added. Fluorescence was measured using a colorimeter at an excitation wavelength of 606 nm. Each experiment was performed in quadruplicate (*n* = 4) to ensure the reproducibility of the results.

### 5.7. Animals

Male Wistar rats weighing 200–250 g and aged 6–8 weeks were maintained on a 12:12 light/dark cycle at 21 ± 2 °C, with ad libitum access to water and food. The experimental groups were formed as follows: (a) intact control group (*n* = 10) with a standard diet (23% protein, 3% fat, and 6% fiber), with ad libitum access to water; and (b) hypercaloric group (*n* = 10) with a high-fat and carbohydrate diet (16% protein, 10% fat, and 2% fiber), with ad libitum access to water and 20% fructose. The weights of the experimental and control animals and their water and food intake were monitored weekly. After completing the experimental cycles, the rats were sacrificed with an overdose of a Zoletil^®^50 (Guadalajara, Mexico) and xylazine cocktail. All animal experiments were approved by the Animal Welfare and Research Ethics Committee of the Autonomous University of Aguascalientes, following Mexican Official Norm NOM-062-ZOO-1999, with authorization code AUT-B-C-1121-077, and ARRIVE guidelines for experimentation and research applications. The animals were obtained from the Autonomous University of Aguascalientes. The sample size (i.e., *n* = 10 per group) was determined based on the minimum number of animals recommended by the institutional ethics committee, considering an acceptable range of error degrees of freedom (DF) in an analysis of variance (ANOVA) [37].

### 5.8. Histopathology

Biological samples (*n* = 6) were obtained after the necropsy, and liver biopsies were analyzed based on their overall appearance, considering factors such as roughness, size, and coloration of the organ. The following stains were applied to the histological sections: (i) hematoxylin/eosin (H&E) for analyzing the histopathology of treated animals, and (ii) Sirius Red for determining the deposition of collagen fibers (type I = red, type III = green) [38].

### 5.9. Biochemical Markers: Liver Function Tests

Blood was collected immediately after euthanasia through cardiac puncture, and each sample (*n* = 6) was centrifuged at 3500 rpm for 5 min to obtain the serum. The parameters used to determine hyperglycemia and hyperlipidemia were glucose, cholesterol, HDL, and triglycerides. Liver function, both in vitro and in vivo, was assessed based on the concentrations of aspartate aminotransferase (AST) and alanine aminotransferase (ALT). The measurements were conducted by spectrophotometry using a Biosystems BTS-350 (Barcelona, Spain). Each experiment was performed in duplicate to ensure the reproducibility of the results.

### 5.10. ELISA Determination of the ACC-1 Enzyme

In a 96-well plate, 200 µL of each sample (*n* = 6) was added to each well, including the supernatants obtained at different induction times and the sera from the animal models with a high-calorie diet. Additionally, 200 µL of carbonate/bicarbonate solution (prepared with 0.0795 g of sodium carbonate and 0.1465 g of sodium bicarbonate dissolved in 50 mL of solution) was added. As a positive control, total lysate from HepG2 cells (RIPA) was used, and as a negative control, the supernatant from the experimental controls was added. The samples were refrigerated for 24 h. Subsequently, they were washed with PBS-Tween 0.025%, 100 µL of skim milk was added, followed by incubation for 30 min at 37 °C. After incubation, washes with PBS-Tween were performed, and the primary antibody against ACC-1 (dilution 1:50, sc-137104) was added, followed by refrigeration for 24 h. After this period, the samples were washed with PBS-Tween, and 100 µL of the secondary antibody anti mouse (dilution 1:1000, Invitrogen 31430) was added. Finally, 100 µL of the detection solution, containing orthophenylenediamine and H_2_O_2_, was added, and the reaction was stopped with H_2_SO_4_. Absorbance was measured at 450 nm. Each experiment was performed in duplicate to ensure the reproducibility of the results.

### 5.11. Quantitative Reverse-Transcription Polymerase Chain Reaction (qRT-PCR)

Total RNA was isolated from both HepG2 cells treated with the previously mentioned high-calorie medium and the tissues of the experimental animals using Direct-zol™ RNA MiniPrep (Zymo Research, Irvine, CA, USA). The cDNA reaction was synthesized with PCR primer pairs designed to amplify 18S rRNA and *acc-1*, *fasn*, *cpt-1*, *acc-2*, *bax*, *bcl-2*, and *caspase-3* (Table 1). Quantitative PCR was performed on the Applied Biosystems StepOne™ Real-Time PCR System using the 2−ΔΔCt method. Relative expression levels were normalized against *18S rRNA* as the internal housekeeping gene. Melting curve analysis was used to verify the specificity of PCR products. Each experiment was performed in sextuplicate (*n* = 6) to ensure the reproducibility of the results.

### 5.12. Statistical Analysis

Statistical analyses were performed using Microsoft Excel and GraphPad Prism version 8.0.1 The results are presented as the mean ± SEM. D’Agostino–Pearson normality test, analysis of variance (ANOVA), and Dunnett’s post hoc test were used for multiple comparisons. *p* values less than 0.05 were considered statistically significant and are indicated by asterisks as follows: * *p* < 0.05, ** *p* < 0.01, *** *p* < 0.001, **** *p* < 0.0001.

## 6. Conclusions

The enzyme ACC-1 could potentially serve as a biomarker, based on both in vitro and in vivo results. The primary membrane damage and histological morphological changes suggest that ACC-1 can be detected at early stages, in contrast to other reference biomarkers such as transaminases ALT and AST. ACC-1 could be prioritized as a biomarker for monitoring patients with hyperlipidemia for the diagnosis of hepatic steatosis or its progression to non-alcoholic steatohepatitis.

## Figures and Tables

**Table 1:** Sequences of the qPCR primers.

Gene	Specie	Forward (5′-3′)	Reverse (5′-3′)
*18SrRNA*	Human	5′-AAA CGG CTA CCA CAT CCA AG-3′	5′-CCT CCA ATG GAT CCT CGT TA-3′
*18SrRNA*	Rat	5′-CGG CTA CCA CAT CCA AGG A-3′	5′-CCA ATT ACA GGG CCT CGA AA-3′
*acc-1*	Human	5′-GGA TGG TGT TCA CTC GGT AAT AGA-3′	5′-GGG TGA TAT GTG CTG CTG CAT-3′
*acc-1*	Rat	5′-TAC AAC GCA GGC ATC AGA AG-3′	5′-TGT GCT GCA GGA AGA TTG AC-3′
*fasn*	Human	5′-CGC TCG GCA TGG CTA TCT-3′	5′-CTG GTT GAA GAA CGC ATC CA-3′
*fasn*	Rat	5′-TCG AGA CAC ATC GTT TGA GC-3′	5′-CCC AGA GGG TGG TTG TTA GA-3′
*cpt-1*	Human	5′-GCA GCG TTC TTT GTG ACG TT-3′	5′-AGG AGT GTT CAG CGT TGA GG-3′
*acc-2*	Human	5’-ACATGGCAAGAGAAAAGCGG-3’	5’-ACTCTTGGTGATCGGCTTGG-3’
*bax*	Human	5′-CGA ACT GGA CAG TAA CAT GGA G-3′	5′-CAG TTT GCT GGC AAA GTA GAA A-3′
*bcl-2*	Human	5′- GAC TTC GCC GAG ATG TCC AG-3′	5′-GAA CTC AAA GAA GGC CAC AAT C-3′
*caspase 3*	Human	5′-TGG AAC CAA AGA TCA TAC ATG G-3′	5′-GTT TGC TGC ATC GAC ATC TG-3′

## Data Availability

The data presented in this study are available upon request from the corresponding author due to privacy.
